# 6-(2-Methoxy­benzyl­amino)purine

**DOI:** 10.1107/S1600536808009203

**Published:** 2008-04-10

**Authors:** Zdeněk Trávníček, Miroslava Matiková-Maľarová, Jiří Mikulík

**Affiliations:** a Department of Inorganic Chemistry, Faculty of Science, Palacký University, Křížkovského 10, CZ-771 47 Olomouc, Czech Republic

## Abstract

The title compound, C_13_H_13_N_5_O, consists of discrete mol­ecules connected by N—H⋯N hydrogen bonds to form infinite chains, with N⋯N separations of 3.0379 (15) and 2.8853 (15) Å. The benzene and purine ring systems make a dihedral angle of 77.58 (3)°. The crystal structure is further stabilized by intra­molecular N⋯O inter­actions [2.9541 (12) Å] and inter­molecular C—H⋯C and C⋯C contacts [3.304 (2), 3.368 (2), 3.667 (2), 3.618 (2) and 3.512 (2) Å] which arrange the mol­ecules into graphite-like layers. The inter­layer separations are 3.248 and 3.256 Å.

## Related literature

For related structures of 6-benzyl­amino­purine derivatives, see: Maloň *et al.* (2001 ▶); Trávníček *et al.* (2006 ▶); Trávníček & Rosenker (2006 ▶). For a description of the Cambridge Structural Database, see: Allen (2002 ▶).
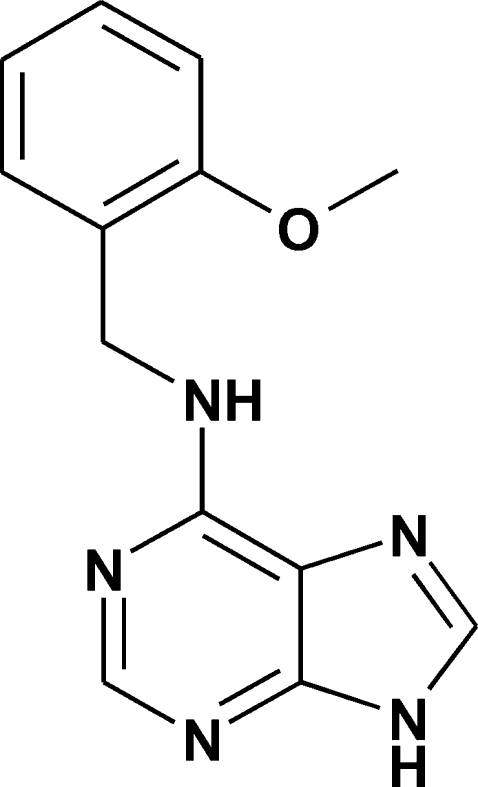


## Experimental

### 

#### Crystal data

C_13_H_13_N_5_O
*M* *_r_* = 255.28
Triclinic, 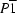

*a* = 7.3518 (2) Å
*b* = 8.0877 (2) Å
*c* = 9.9771 (3) Å
α = 78.439 (3)°
β = 85.099 (2)°
γ = 83.803 (2)°
*V* = 576.56 (3) Å^3^
*Z* = 2
Mo *K*α radiation radiation
μ = 0.10 mm^−1^
*T* = 120 (2) K
0.20 × 0.20 × 0.15 mm

#### Data collection

Oxford Diffraction Xcalibur2 diffractometer
Absorption correction: multi-scan (*CrysAlis RED*; Oxford Diffraction, 2007 ▶) *T*_min_ = 0.947, *T*_max_ = 0.990
4904 measured reflections
2026 independent reflections
1709 reflections with *I* > 2σ(*I*)
*R*_int_ = 0.018

#### Refinement

*R*[*F* ^2^ > 2σ(*F* ^2^)] = 0.030
*wR*(*F* ^2^) = 0.082
*S* = 1.09
2026 reflections
173 parameters
H-atom parameters constrained
Δρ_max_ = 0.20 e Å^−3^
Δρ_min_ = −0.20 e Å^−3^

### 

Data collection: *CrysAlis CCD* (Oxford Diffraction, 2007 ▶); cell refinement: *CrysAlis RED* (Oxford Diffraction, 2007 ▶); data reduction: *CrysAlis RED*; program(s) used to solve structure: *SHELXS97* (Sheldrick, 2008 ▶); program(s) used to refine structure: *SHELXL97* (Sheldrick, 2008 ▶); molecular graphics: *DIAMOND* (Brandenburg, 2006 ▶); software used to prepare material for publication: *SHELXL97* and *DIAMOND*.

## Supplementary Material

Crystal structure: contains datablocks I, global. DOI: 10.1107/S1600536808009203/bh2166sup1.cif

Structure factors: contains datablocks I. DOI: 10.1107/S1600536808009203/bh2166Isup2.hkl

Additional supplementary materials:  crystallographic information; 3D view; checkCIF report

## Figures and Tables

**Table 1:** Hydrogen-bond geometry (Å, °)

*D*—H⋯*A*	*D*—H	H⋯*A*	*D*⋯*A*	*D*—H⋯*A*
N6—H6*A*⋯N7^i^	0.88	2.19	3.0379 (15)	162
N9—H9*C*⋯N3^ii^	0.88	2.02	2.8853 (15)	167
C16—H16*C*⋯C14^iii^	0.98	2.87	3.6666 (18)	139
C16—H16*B*⋯C15^iv^	0.98	2.85	3.6182 (18)	136
C12—H12*A*⋯C6^iii^	0.95	2.77	3.5119 (17)	136

Symmetry codes: (i) 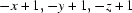; (ii) 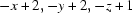; (iii) 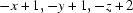; (iv) 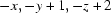.

**Fig. 1.**
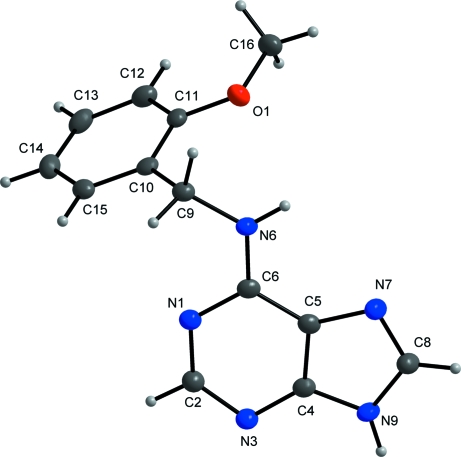
The molecular structure of (I). Non-H atoms are drawn with 50% probability displacement ellipsoids.

**Fig. 2.**
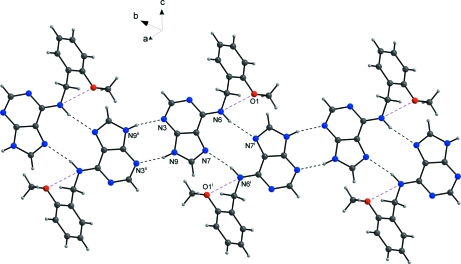
Part of the crystal structure of (I), showing the formation of infinite chains, N—H···N hydrogen bonds [N6···N7i = 3.0379 (15) Å, N9···N3ii = 2.8853 (15) Å] and O···N non-bonding contacts [N6···O1 = 2.9541 (12) Å] (dashed lines). Symmetry codes: (i) 1 - x, 1 - y, -z + 1; (ii) 2 - x, 2 - y, -z + 1.

**Fig. 3.**
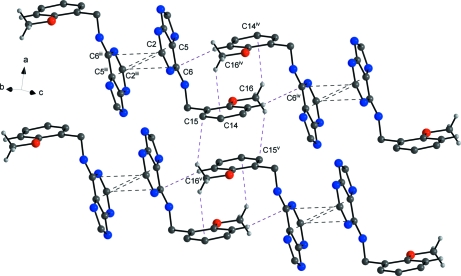
Part of the crystal structure of (I), showing the C···C and C—H···C interactions (dashed lines) connecting molecules among layers. H-atoms not involved into hydrogen bonding have been omitted for clarity. Symmetry codes: (iii) 1 - x, 2 - y, 1 - z; (iv) 1 - x, 1 - y, 2 - z; (v) -x, 1 - y, 2 - z.

**Fig. 4.**
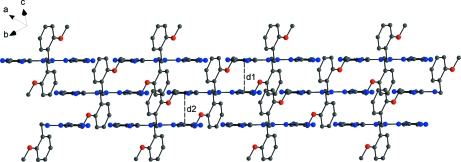
Part of the crystal structure of (I), showing the formation of graphite-like layers. Dashed lines represent the shortest distances between two neighbouring layers formed by purine moieties (d1 = 3.256, d2 = 3.248 Å). H-atoms have been omitted for clarity.

## supplementary crystallographic information

### Comment

The structure of the title molecule, (I), extends our crystallographic knowledge regarding aromatic cytokinins and cyclin dependent kinase inhibitors derived from 6-benzylaminopurine.

The molecular structure of (I) is shown in Fig. 1. The molecule contains three different aromatic rings: benzene (A), pyrimidine (B) and imidazole (C). Each ring is essentially planar with the maximum deviations from the least-squares planes being 0.0169 (12) Å for C11 (ring A), 0.0147 (12) Å for C6 (ring B), and 0.0054 (13) Å for C8 (ring C). The dihedral angle between benzene ring (A) and purine skeleton (rings B and C) is 77.58 (3)°, whilst the pyrimidine (B) and imidazole (C) rings are almost coplanar, making a dihedral angle of 3.84 (4)° (Brandenburg, 2006). The interatomic parameters of (I) are comparable to those found for compounds bearing an electroneutral N9—H 6-benzylaminopurine moiety, *e.g*. 6-(2-chlorobenzylamino)purine dihydrate (Maloň *et al.*, 2001), 6-(2-bromobenzylamino)purine (Trávníček & Rosenker, 2006) and 6-(2-chloro-4-fluorobenzylamino)purine (Trávníček *et al.*, 2006). To date, 59 structures of compounds involving the 6-benzylaminopurine skeleton have been deposited in the CSD (Cambridge Structural Database, Version 5.29; Allen, 2002).

The secondary structure of (I) is stabilized by intermolecular hydrogen bonds of the N—H···N type (Table 1, Fig. 2), which connect the molecules into infinite one-dimensional chains. Moreover, intramolecular N···O interactions [N6···O1 = 2.9541 (12) Å, Fig. 2], and non-bonding intermolecular interactions of the type C···C [C2···C5^iii^ = 3.304 (2) Å, C2···C6^iii^ = 3.368 (2) Å] and C—H···C [C16···C14^iv^ = 3.667 (2), C16···C15^v^ = 3.618 (2), and C12···C6^iv^ = 3.512 (2) Å; symmetry codes: (iii) 1 - *x*, 2 - *y*, 1-*z;* (iv) 1 - *x*, 1 - *y*, 2-*z;* (v) -*x*, 1 - *y*, 2 - *z*] also contribute to the stabilization of the crystal structure (Fig. 3). The later non-bonding interactions arrange the molecules into *graphite-like* layers (Fig. 4). The separations between two layers formed by purine moieties are not equal, with the shortest distances being 3.248 and 3.256 Å. For comparison, the corresponding layer-to-layer separation has been found to be 3.352 Å (Space group *P*6_3_/*mmc*, ICSD No. 52230), and 3.395 Å (Space group *P*6_3_*mc*, ICSD No. 31170) in the crystal structure of graphite, as deposited in the ICSD (The Inorganic Crystal Structure Database, Version 1.4.2, 2007–2 and calculated using *DIAMOND* (Brandenburg, 2006).

### Experimental

The title compound, was synthesized by a recently described method (Trávníček & Rosenker, 2006). The obtained microcrystalline product was recrystallized from hot *N*,*N*-dimethylformamide. Well shaped colourless single crystals, suitable for X-ray structural analysis, were formed after slow evaporation of the solvent over a period of few days. The crystals were filtered off, washed with EtOH and Et_2_O and dried in air.

### Refinement

All H atoms were located in difference maps and refined using a riding model, with C—H distances fixed to 0.95 (CH) or 0.98 (CH_3_) Å, N—H distances to 0.88 Å, and with *U*_iso_(H) values of 1.2*U*_eq_(CH, N) or 1.5*U*_eq_(CH_3_). The highest unassigned difference Fourier peak, 0.198 e.Å^-3^, is located at 0.24 Å from atom H16A.

### Crystal data

**Table:**

C_13_H_13_N_5_O	*Z* = 2
*M**_r_* = 255.28	*F*_000_ = 268
Triclinic, *P*1	*D*_x_ = 1.470 Mg m^−^^3^
Hall symbol: -P 1	Mo *K*α radiation λ = 0.71073 Å
*a* = 7.3518 (2) Å	Cell parameters from 4019 reflections
*b* = 8.0877 (2) Å	θ = 2.8–31.9º
*c* = 9.9771 (3) Å	µ = 0.10 mm^−^^1^
α = 78.439 (3)º	*T* = 120 (2) K
β = 85.099 (2)º	Prism, colourless
γ = 83.803 (2)º	0.20 × 0.20 × 0.15 mm
*V* = 576.56 (3) Å^3^	

### Data collection

**Table:**

Oxford Diffraction Xcalibur2 diffractometer	2026 independent reflections
Radiation source: Enhance (Mo) X-ray Source	1709 reflections with *I* > 2σ(*I*)
Monochromator: graphite	*R*_int_ = 0.018
Detector resolution: 8.3611 pixels mm^-1^	θ_max_ = 25.0º
*T* = 120(2) K	θ_min_ = 2.8º
rotation method, ω scans	*h* = −8→6
Absorption correction: multi-scan(CrysAlis RED; Oxford Diffraction, 2007)	*k* = −9→9
*T*_min_ = 0.947, *T*_max_ = 0.990	*l* = −11→11
4904 measured reflections	

### Refinement

**Table:**

Refinement on *F*^2^	Secondary atom site location: difference Fourier map
Least-squares matrix: full	Hydrogen site location: inferred from neighbouring sites
*R*[*F*^2^ > 2σ(*F*^2^)] = 0.030	H-atom parameters constrained
*wR*(*F*^2^) = 0.082	*w* = 1/[σ^2^(*F*_o_^2^) + (0.0452*P*)^2^ + 0.0986*P*] where *P* = (*F*_o_^2^ + 2*F*_c_^2^)/3
*S* = 1.09	(Δ/σ)_max_ < 0.001
2026 reflections	Δρ_max_ = 0.20 e Å^−^^3^
173 parameters	Δρ_min_ = −0.20 e Å^−^^3^
Primary atom site location: structure-invariant direct methods	Extinction correction: none

### Special details

**Table:**

Experimental. empirical absorption correction using spherical harmonics implemented in SCALE3 ABSPACK scaling algorithm.

### Fractional atomic coordinates and isotropic or equivalent isotropic displacement parameters (Å^2^)

**Table:**

	*x*	*y*	*z*	*U*_iso_*/*U*_eq_	
O1	0.27430 (12)	0.38665 (11)	0.87665 (9)	0.0239 (2)	
N1	0.47080 (14)	0.91080 (13)	0.69756 (10)	0.0198 (3)	
C2	0.61005 (17)	1.00797 (16)	0.68033 (13)	0.0212 (3)	
H2A	0.5924	1.1014	0.7260	0.025*	
N3	0.77035 (14)	0.99274 (13)	0.60829 (10)	0.0203 (3)	
C4	0.77955 (16)	0.86036 (15)	0.54352 (12)	0.0179 (3)	
C5	0.64716 (17)	0.74864 (15)	0.55142 (12)	0.0184 (3)	
C6	0.48638 (16)	0.77580 (15)	0.63459 (12)	0.0178 (3)	
N6	0.34980 (14)	0.67530 (13)	0.65272 (10)	0.0196 (3)	
H6A	0.3624	0.5856	0.6142	0.023*	
N7	0.69973 (14)	0.63329 (14)	0.46629 (10)	0.0232 (3)	
C8	0.85956 (18)	0.67833 (17)	0.41063 (13)	0.0247 (3)	
H8A	0.9298	0.6219	0.3459	0.030*	
N9	0.91613 (14)	0.81253 (13)	0.45352 (10)	0.0210 (3)	
H9C	1.0196	0.8591	0.4284	0.025*	
C9	0.18057 (16)	0.70950 (16)	0.73431 (12)	0.0196 (3)	
H9A	0.0868	0.6409	0.7134	0.024*	
H9B	0.1357	0.8304	0.7060	0.024*	
C10	0.19889 (16)	0.67198 (15)	0.88728 (12)	0.0178 (3)	
C11	0.24921 (16)	0.50695 (15)	0.95637 (12)	0.0189 (3)	
C12	0.27108 (17)	0.47421 (17)	1.09645 (13)	0.0233 (3)	
H12A	0.3090	0.3628	1.1424	0.028*	
C13	0.23723 (17)	0.60508 (18)	1.16864 (13)	0.0252 (3)	
H13A	0.2540	0.5831	1.2642	0.030*	
C14	0.17943 (17)	0.76710 (17)	1.10332 (13)	0.0244 (3)	
H14A	0.1515	0.8554	1.1539	0.029*	
C15	0.16273 (16)	0.79907 (16)	0.96262 (13)	0.0208 (3)	
H15A	0.1256	0.9109	0.9171	0.025*	
C16	0.32229 (18)	0.21625 (16)	0.94300 (14)	0.0277 (3)	
H16A	0.3315	0.1419	0.8758	0.042*	
H16B	0.2278	0.1808	1.0154	0.042*	
H16C	0.4405	0.2089	0.9834	0.042*	

### Atomic displacement parameters (Å^2^)

**Table:**

	*U*^11^	*U*^22^	*U*^33^	*U*^12^	*U*^13^	*U*^23^
O1	0.0292 (5)	0.0175 (5)	0.0249 (5)	−0.0026 (4)	−0.0002 (4)	−0.0043 (4)
N1	0.0205 (6)	0.0192 (6)	0.0199 (5)	−0.0039 (4)	−0.0013 (4)	−0.0033 (4)
C2	0.0225 (7)	0.0211 (7)	0.0209 (7)	−0.0036 (5)	−0.0015 (5)	−0.0050 (5)
N3	0.0201 (6)	0.0212 (6)	0.0203 (6)	−0.0046 (4)	−0.0015 (4)	−0.0046 (4)
C4	0.0187 (6)	0.0191 (6)	0.0153 (6)	−0.0034 (5)	−0.0034 (5)	−0.0003 (5)
C5	0.0225 (6)	0.0178 (6)	0.0149 (6)	−0.0040 (5)	−0.0034 (5)	−0.0009 (5)
C6	0.0205 (6)	0.0186 (6)	0.0137 (6)	−0.0038 (5)	−0.0039 (5)	0.0008 (5)
N6	0.0214 (6)	0.0198 (6)	0.0188 (5)	−0.0079 (4)	0.0017 (4)	−0.0046 (4)
N7	0.0262 (6)	0.0247 (6)	0.0207 (6)	−0.0082 (5)	0.0033 (5)	−0.0081 (5)
C8	0.0270 (7)	0.0255 (7)	0.0239 (7)	−0.0096 (6)	0.0044 (5)	−0.0088 (6)
N9	0.0196 (6)	0.0240 (6)	0.0204 (6)	−0.0084 (4)	0.0021 (4)	−0.0049 (4)
C9	0.0181 (6)	0.0192 (6)	0.0218 (7)	−0.0042 (5)	−0.0016 (5)	−0.0031 (5)
C10	0.0113 (6)	0.0214 (6)	0.0208 (7)	−0.0052 (5)	0.0009 (5)	−0.0030 (5)
C11	0.0137 (6)	0.0211 (7)	0.0225 (7)	−0.0049 (5)	0.0027 (5)	−0.0058 (5)
C12	0.0198 (7)	0.0254 (7)	0.0225 (7)	−0.0026 (5)	−0.0010 (5)	0.0010 (5)
C13	0.0201 (7)	0.0373 (8)	0.0185 (7)	−0.0057 (6)	0.0013 (5)	−0.0057 (6)
C14	0.0204 (7)	0.0299 (7)	0.0260 (7)	−0.0050 (5)	0.0016 (5)	−0.0130 (6)
C15	0.0156 (6)	0.0201 (7)	0.0267 (7)	−0.0033 (5)	0.0002 (5)	−0.0044 (5)
C16	0.0259 (7)	0.0186 (7)	0.0366 (8)	−0.0018 (5)	0.0039 (6)	−0.0032 (6)

### Geometric parameters (Å, °)

**Table:**

O1—C11	1.3636 (15)	N9—H9C	0.8800
O1—C16	1.4252 (15)	C9—C10	1.5100 (17)
N1—C2	1.3359 (16)	C9—H9A	0.9900
N1—C6	1.3549 (16)	C9—H9B	0.9900
C2—N3	1.3342 (16)	C10—C15	1.3815 (17)
C2—H2A	0.9500	C10—C11	1.4007 (17)
N3—C4	1.3500 (16)	C11—C12	1.3893 (18)
C4—N9	1.3659 (15)	C12—C13	1.3853 (19)
C4—C5	1.3850 (17)	C12—H12A	0.9500
C5—N7	1.3874 (16)	C13—C14	1.3810 (19)
C5—C6	1.4083 (17)	C13—H13A	0.9500
C6—N6	1.3371 (16)	C14—C15	1.3891 (19)
N6—C9	1.4594 (16)	C14—H14A	0.9500
N6—H6A	0.8800	C15—H15A	0.9500
N7—C8	1.3121 (17)	C16—H16A	0.9800
C8—N9	1.3594 (17)	C16—H16B	0.9800
C8—H8A	0.9500	C16—H16C	0.9800
			
C11—O1—C16	117.32 (10)	N6—C9—H9B	108.6
C2—N1—C6	118.43 (11)	C10—C9—H9B	108.6
N3—C2—N1	129.36 (12)	H9A—C9—H9B	107.6
N3—C2—H2A	115.3	C15—C10—C11	118.51 (11)
N1—C2—H2A	115.3	C15—C10—C9	120.72 (11)
C2—N3—C4	110.81 (10)	C11—C10—C9	120.75 (11)
N3—C4—N9	127.62 (11)	O1—C11—C12	124.23 (11)
N3—C4—C5	126.45 (12)	O1—C11—C10	115.33 (11)
N9—C4—C5	105.89 (11)	C12—C11—C10	120.43 (12)
C4—C5—N7	110.49 (11)	C13—C12—C11	119.59 (12)
C4—C5—C6	117.01 (11)	C13—C12—H12A	120.2
N7—C5—C6	132.41 (11)	C11—C12—H12A	120.2
N6—C6—N1	119.26 (11)	C14—C13—C12	120.74 (12)
N6—C6—C5	122.86 (11)	C14—C13—H13A	119.6
N1—C6—C5	117.88 (11)	C12—C13—H13A	119.6
C6—N6—C9	122.18 (10)	C13—C14—C15	119.07 (12)
C6—N6—H6A	118.9	C13—C14—H14A	120.5
C9—N6—H6A	118.9	C15—C14—H14A	120.5
C8—N7—C5	103.37 (10)	C10—C15—C14	121.55 (12)
N7—C8—N9	114.30 (11)	C10—C15—H15A	119.2
N7—C8—H8A	122.8	C14—C15—H15A	119.2
N9—C8—H8A	122.8	O1—C16—H16A	109.5
C8—N9—C4	105.94 (10)	O1—C16—H16B	109.5
C8—N9—H9C	127.0	H16A—C16—H16B	109.5
C4—N9—H9C	127.0	O1—C16—H16C	109.5
N6—C9—C10	114.61 (10)	H16A—C16—H16C	109.5
N6—C9—H9A	108.6	H16B—C16—H16C	109.5
C10—C9—H9A	108.6		

### Hydrogen-bond geometry (Å, °)

**Table:**

*D*—H···*A*	*D*—H	H···*A*	*D*···*A*	*D*—H···*A*
N6—H6A···N7^i^	0.88	2.19	3.0379 (15)	162
N9—H9C···N3^ii^	0.88	2.02	2.8853 (15)	167
C16—H16C···C14^iii^	0.98	2.87	3.6666 (18)	139
C16—H16B···C15^iv^	0.98	2.85	3.6182 (18)	136
C12—H12A···C6^iii^	0.95	2.77	3.5119 (17)	136

Symmetry codes: (i) −*x*+1, −*y*+1, −*z*+1; (ii) −*x*+2, −*y*+2, −*z*+1; (iii) −*x*+1, −*y*+1, −*z*+2; (iv) −*x*, −*y*+1, −*z*+2.
